# Ulcers and Wounds

**Published:** 1872-11

**Authors:** 


### ULCERS AND WOUNDS.

WASH twice a day with one drachm of carbolic acid in eight ounces of water; it is the best remedy known. At the same time let it be distinctly understood that it is never safe to heal up any sore, or to make any external applications to eruptions or breakings out on the skin, unless the bowels are made to act freely at least once every day, and if twice, so much the better, for the parts will get well sooner.

